# Integrating vectors for genetic studies in the rare Actinomycete *Amycolatopsis marina*

**DOI:** 10.1186/s12896-019-0521-y

**Published:** 2019-06-04

**Authors:** Hong Gao, Buvani Murugesan, Janina Hoßbach, Stephanie K. Evans, W. Marshall Stark, Margaret C. M. Smith

**Affiliations:** 1 Department of Biology, University of York, York, North Yorkshire YO10 5DD UK; 2 Institute of Molecular, Cell and Systems Biology, University of Glasgow, Glasgow, G12 8QQ UK; 3 Present address: School of Science, Engineering & Design, Teesside University, Middlesbrough, TS1 3BX UK

**Keywords:** Rare Actinomycetes, *Amycolatopsis*, Integrating vectors, TG1 integrase, R4 integrase

## Abstract

**Background:**

Few natural product pathways from rare Actinomycetes have been studied due to the difficulty in applying molecular approaches in these genetically intractable organisms. In this study, we sought to identify more integrating vectors, using phage *int/attP* loci, that would efficiently integrate site-specifically in the rare Actinomycete, *Amycolatopsis marina* DSM45569.

**Results:**

Analysis of the genome of *A. marina* DSM45569 indicated the presence of *attB*-like sequences for TG1 and R4 integrases. The TG1 and R4 *attB*s were active in in vitro recombination assays with their cognate purified integrases and *attP* loci. Integrating vectors containing either the TG1 or R4 *int/attP* loci yielded exconjugants in conjugation assays from *Escherichia coli* to *A. marina* DSM45569. Site-specific recombination of the plasmids into the host TG1 or R4 *attB* sites was confirmed by sequencing.

**Conclusions:**

The homologous TG1 and R4 *attB* sites within the genus *Amycolatopsis* have been identified. The results indicate that vectors based on TG1 and R4 integrases could be widely applicable in this genus.

## Background

*Streptomyces* bacteria are widely exploited for their abundant bioactive natural products [1]. However, after decades of exploitation, the rate of discovery of new *Streptomyces*-derived bioactive products has declined, and interest has grown in other potential non-Streptomycete sources, such as the rare Actinomycetes [2, 3].

Amongst rare Actinomycetes, the *Amycolatopsis* genus is of particular interest for its production of critically important antibiotics such as vancomycin [4] and rifamycin [5], as well as a diverse range of active natural products [6–8]. The publicly available NCBI database contains nearly 90 genomes of *Amycolatopsis* strains, covering more than 40 species from this genus. Similar to *Streptomyces*, the genome of each *Amycolatopsis* contains averagely over 20 secondary metabolic gene clusters [9]. The mining of these metabolic clusters offers excellent potential for novel antibiotic discovery.

Phage-encoded serine and tyrosine integrases catalyse site-specific integration of a circularised phage genome into the host chromosome as part of the process to establish a lysogen. DNA integration mediated by serine integrases occurs between short (approximately 50 bp) DNA substrates that are located on the phage genome (the phage attachment site *attP*), and the host genome (the bacterial attachment site *attB*). The product of *attP* x *attB* recombination is an integrated phage genome flanked by two new sites, *attL* and *attR*, each of which contains half-sites from *attP* and *attB*. During phage induction, integrase in the presence of a recombination directionality factor (RDF) again mediates site-specific recombination, but this time between *attL* and *attR*, to excise the phage genome, which can then be replicated during a lytic cycle. The mechanism of recombination and the factors that control integration versus excision have been elucidated in recent years [10–12].

Integrating vectors based on the *Streptomyces* phage ϕC31 integrase and *attP* locus are best known and most widely used in Actinomycete genome engineering [13–16], and in addition to the phage recombination machinery (*int/attP*), integrating vectors contain a replicon for maintenance in *Escherichia coli*, an *oriT* for conjugal transfer and a marker or markers for selection in *E. coli* and the recipient. They are powerful genome engineering tools that act in an efficient, highly controllable and predictable way [17].

Using serine integrase-mediated recombination, these integrating vectors require no additional phage or host functions for integration, which is an especially important feature when they are used in other organisms that cannot be infected by the phages. This property makes serine integrase-based vectors promising tools for use in various systems [10, 18]. However, the use of these integration vectors has not been fully explored in rare Actinomycetes, e.g. *Amycolatopsis*. There is one reported example of a conjugation system based on ϕC31 integrase in *Amycolatopsis japonicum* MG417-CF17 [19], and it has been reported that other *Amycolatopsis* species lack ϕC31 *attB* sites in their chromosomes [20]. The ϕBT1 *attB* sites have been more commonly identified in *Amycolatopsis*. A vector based on ϕBT1 *int/attP* has been successfully transferred into *Amycolatopsis mediterranei* [21]. Furthermore, electroporation remains the most widely applied method for transfer of integrative plasmids into this genus, rather than conjugation [20, 21].

In this paper, we chose to study *A. marina* DSM45569, a species isolated from an ocean-sediment sample collected in the South China Sea [22]. Since the marine environment has been assumed to offer an as yet mostly untapped treasure of chemical biodiversity [23], we are quite interested in natural product discovery from *A. marina*. We explored the application of bacterial genetic engineering using serine integrases and developed conjugative and integrating vectors for use in this species. We present evidence suggesting that these vectors could be applied to other species in this genus, thus opening up the prospect for versatile genetic manipulation of *Amycolatopsis*.

## Results

### Identification of *attB*-like sequences from the genome of *A. marina* DSM45569

**Fig. 1:**
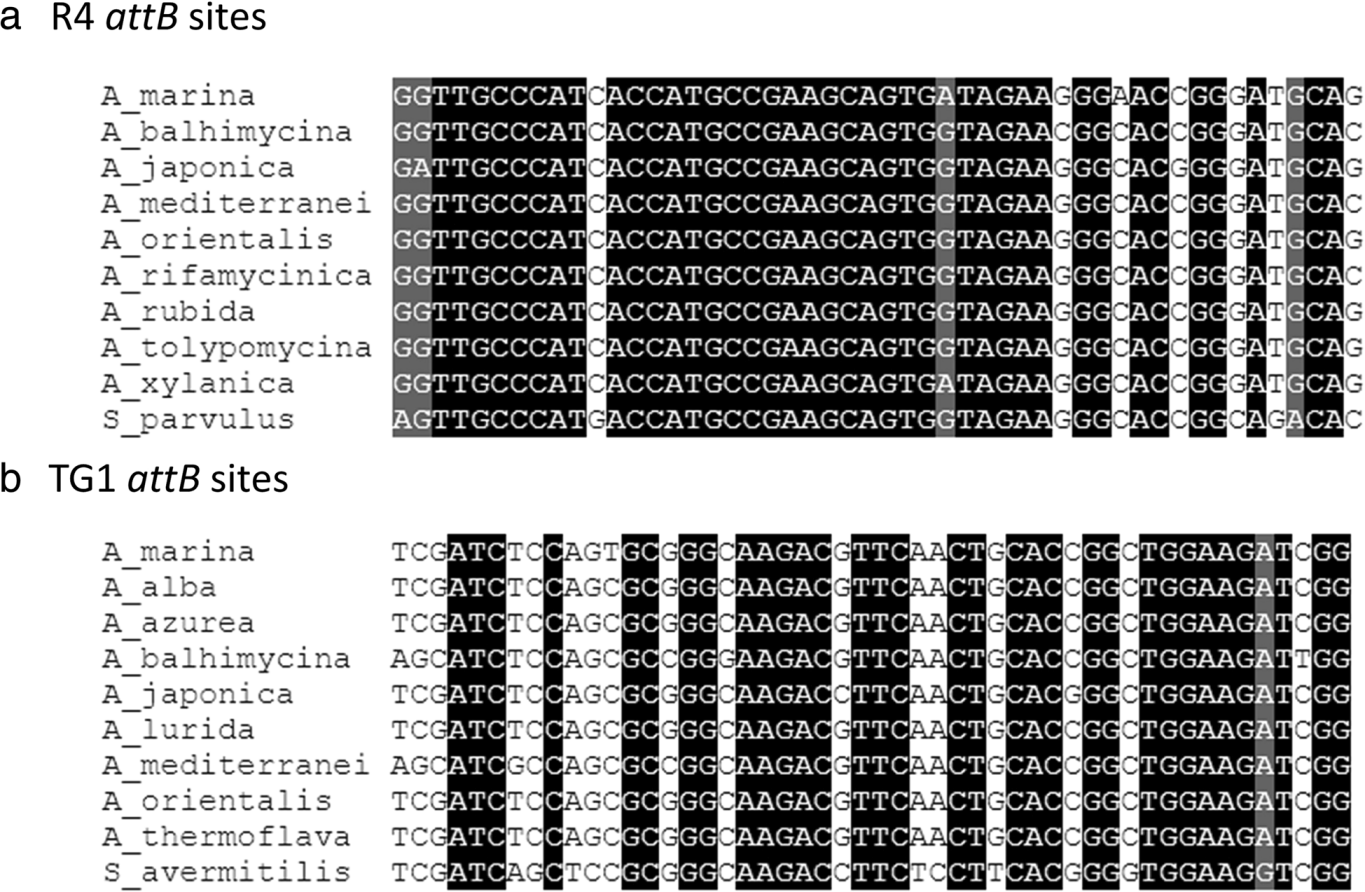
Alignment of R4 and TG1 *attB* sites in *A. marina* DSM45569 and other *Amycolatopsis* species. **a**) GenBank accession nos. of DNA sequences: *Amycolatopsis balhimycina* (ARBH01000005.1), *Amycolatopsis japonica* (NZ_CP008953.1), *Amycolatopsis mediterranei* (NC_022116.1), *Amycolatopsis orientalis* (NZ_CP016174.1), *Amycolatopsis rifamycinica* (NZ_JMQI01000006.1), *Amycolatopsis rubida* (NZ_FOWC01000001.1), *Amycolatopsis tolypomycina* (NZ_FNSO01000004.1), *Amycolatopsis xylanica* (NZ_FNON01000002.1), and *S. parvulus* (CP015866.1); **b**) GenBank accession nos. of DNA sequences: *Amycolatopsis alba* (NZ_KB913032.1), *Amycolatopsis azurea* (MUXN01000005.1), *A. balhimycina* (ARBH01000007.1), *A. japonica* (NZ_CP008953.1), *Amycolatopsis lurida* (FNTA01000004.1), *A. mediterranei* (NC_022116.1), *A. orientalis* (NZ_CP01674.1), *Amycolatopsis thermoflava* (AXBH01000004.1), and *S. avermitilis* (NC_003155.5)

The primers used in this study were listed in Table 1. The sequences of *attB* sites recognised by a variety of integrases (φC31 [24], φJoe [25], Bxb1 [26], R4 [27], SPBc [28], SV1 [29], TG1 [30] and TP901 [31]) were used in BLAST searches of the genome sequence of *A. marina* DSM45569 (NCBI Genome Database NZ_FOKG00000000). The most significant hits for R4 and TG1 *attB* sites had the highest identities and lowest *E*-value (Table 2). The recognised R4 *attB-*like site is located within a gene predicted to encode a fatty-acyl-CoA synthase (SFB62308.1), and the TG1 *attB* site is located within a gene predicted to code for a putative succinyldiaminopimelate transaminase (WP_091671332.1). The BLAST analysis was extended to other species of *Amycolatopsis* to assess the conservation of these *attB* sites in the genus (Fig. 1). Both R4 and TG1 *attB* sites were highly conserved relative to the *attB* sites originally identified from *Streptomyces parvulus* [32] (84% for R4 integrase) and *Streptomyces avermitilis* [30] (62% for TG1 integrase).

**Table 1:** Oligonucleotides used in this study

Oligonucleotide	Sequence (5′-3′)
pHG1A-for	CGAACGCATCGATTAATTAAGGAGGATCGTATGACGACCGTTCCCG
pHG1A-rev	CGTGGTGGGCGCTAGCCTCCTCTAGTCATCCGTCG
pHG1-for	ACTAGAGGAGGCTAGCTTCAATGGAGGAGATGATCGAGG
pHG1-rev	GCAGGTCGACTCTAGATCTCGCTACGCCGCTACG
pHG4-for	CGAACGCATCGATTAATTAAGCGGCCGCCATATGGAATTCGGTACCGCATGCAGATCTAGGAACTTCGAAGTTCCCGC
pHG4-rev	TGATTACGCCAAGCTTTCGACTCTAGAGTAAGCGTCACGG
pJH1R4-for	CTAGCGATTGCCATGACGTCGGAGCTGCTTACCAATGTC
pJH1R4-rev	AAGAGGCCCGCACCGATTCCAAGAGGCCGGCAACTAC
TG1-attB-Am-for	TCGATCTCCAGTGCGGGCAAGACGTTCAACTGCACCGGCTGGAAGATCGGGACCACCGGACGAACGCA
TG1-attB-Sa-for	TCGATCAGCTCCGCGGGCAAGACCTTCTCCTTCACGGGGTGGAAGGTCGGCGGTGGAGCTCGGAGA
R4-attB-Am-for	GGTTGCCCATCACCATGCCGAAGCAGTGATAGAAGGGAACCGGGATGCAGGTGAGAAGGTGCTCGTGT
R4-attB-Sp-for	AGTTGCCCATGACCATGCCGAAGCAGTGGTAGAAGGGCACCGGCAGACACGGTGAGAAGGTGCTCGTGT
attB-rev	CTGCATCTCAACGCCTTCCGG
TG1-attP-for	AACCTTCACGCTCATGCC
TG1-attP-rev	GTCGAGATTCTCCGTCTCCTG
R4-attP-for	GATCGGTCTTGCCTTGCTC
R4-attP-rev	ACCCGCAGAGTGTACCCA
TG1-attL-Am-for	ACAACCCCACCGGCACCGTCTTCA
TG1-attL-Am-rev	AGTATAGGAACTTCGAAGCAGCTC
R4-attL-Am-for	CGGCCGGTGATGTTGACGT
R4-attL-Am-rev	TCGGCCGTCACGATGGTCA

The *attB* sequences are shown underlined

**Table 2:**
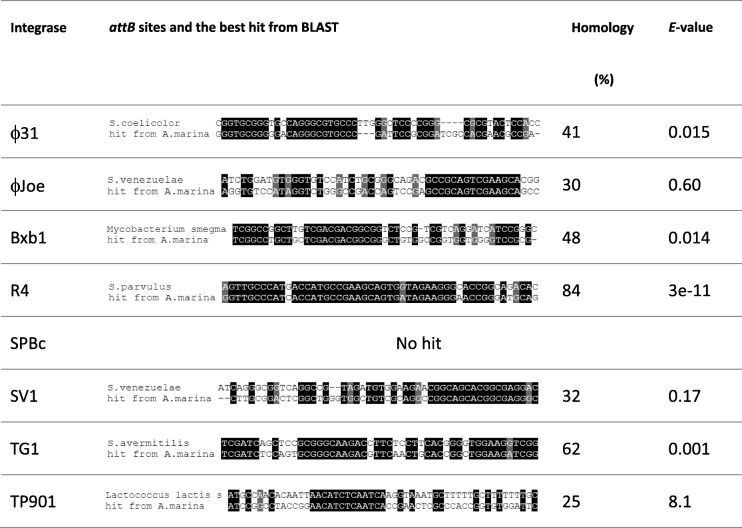
The original *attB* sites for integrases and results of BLAST search

### *A. marina attB*-like sequences for TG1 and R4 are both active in in vitro recombination

**Fig. 2:**
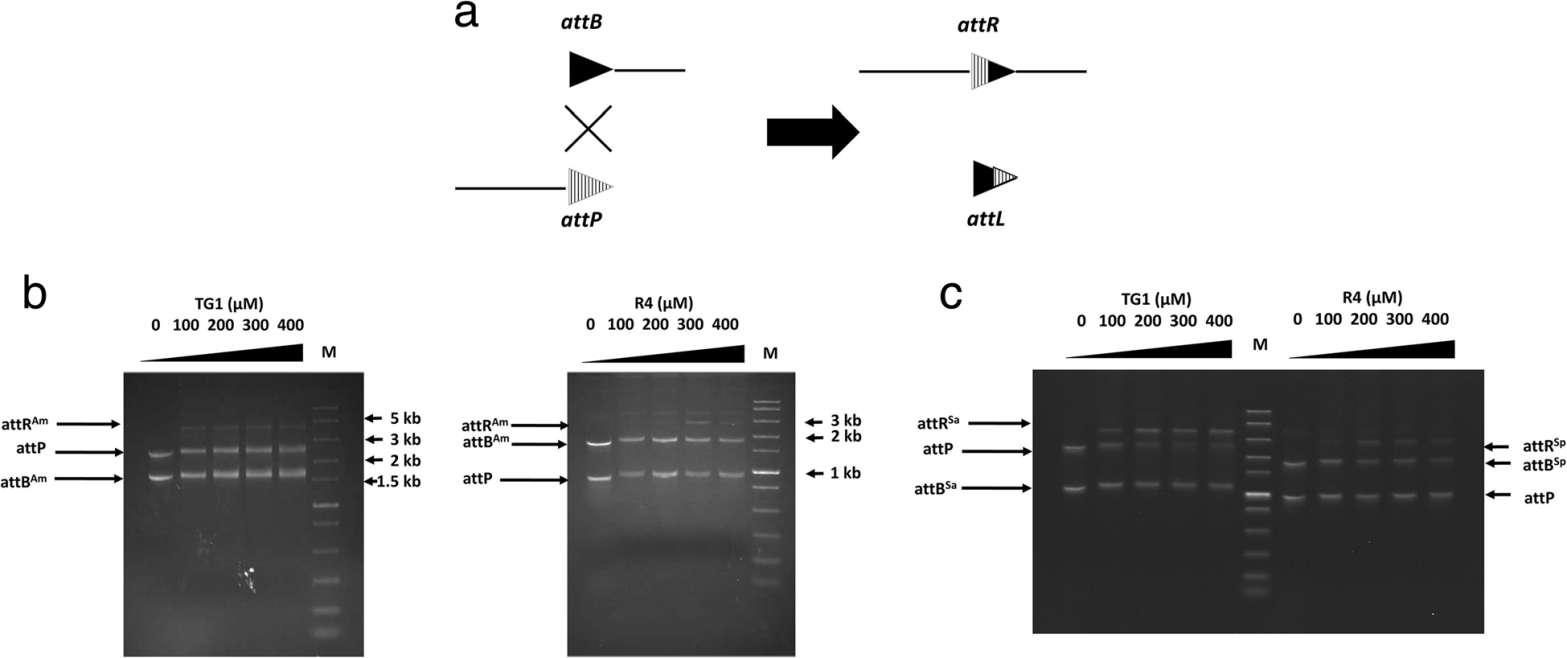
In vitro recombination. (**a**) Recombination substrates and their expected products. (**b**) In vitro recombination between DNA fragments containing TG1 *attB*^*Am*^ (1627 bp) and TG1 *attP* (2471 bp; left), and R4 *attB*^*Am*^ (1854 bp) and R4 *attP* (990 bp; right). The expected products of the TG1 integrase-mediated reaction were a 4.1 kb DNA fragment containing the *attR*^*Am*^ site, and a 53 bp fragment containing *attL*^*Am*^ (not observed). For the R4 integrase recombination reaction, the expected products were a 2.8 kb fragment containing *attR*^*Am*^, and a 51 bp *attL*^*Am*^ fragment (not observed). (**c**) In vitro recombination between DNA fragments containing TG1 *attB*^*Sa*^ (1035 bp) and TG1 *attP* (2471 bp; left), and R4 *attB*^*Sp*^ (1855 bp) and R4 *attP* (990 bp; right). The expected products were a 3.5 kb fragment containing *attR*^*Sa*^ for the TG1 reaction, and a 2.8 kb fragment containing *attR*^*Sp*^ for the R4 reaction. M: Fast DNA Ladder (NEB, USA)

In each recombination reaction, substrates containing *attP* and the putative *attB* site were mixed in cognate pairs with different concentrations of purified R4 or TG1 integrase in the corresponding buffer and incubated overnight at 30 °C, as described in Methods. The expected recombination events and the nature of the products are shown in Fig. 2a. TG1 catalysed recombination between the substrates more efficiently than R4 (Fig. 2b). As expected because neither phage is an *Amycolatopsis* phage, the recombination efficiencies for each integrase were observably better when the *Streptomyces attB* sites were used (Fig. 2c) compared to the *A. marina attB* sites (Fig. 2b), particularly for TG1 integrase. Nevertheless, the presence of recombination activity indicated that both *A. marina attB* sites were functional and were likely to be active integration sites for integrative conjugation vectors.

### In vivo integration

**Fig. 3:**
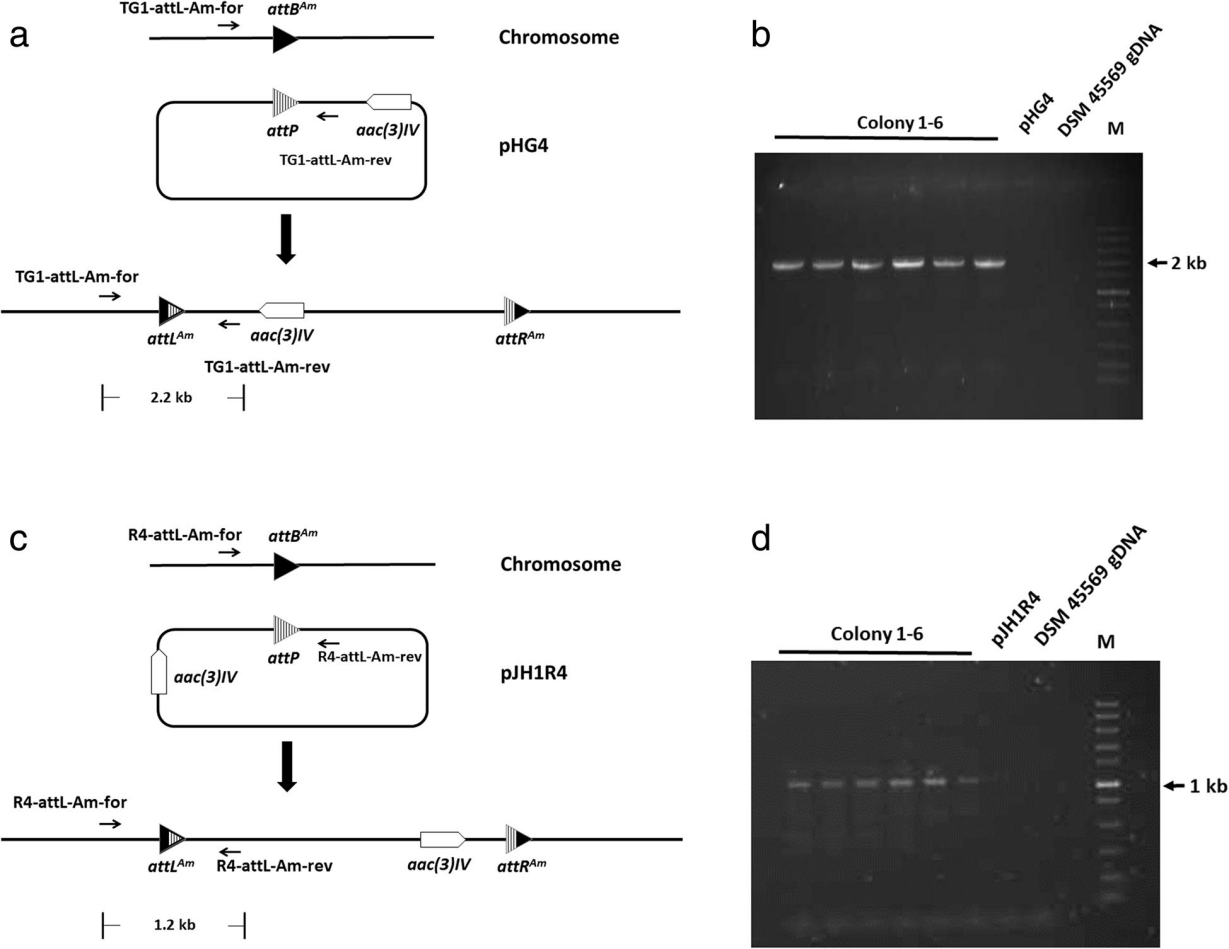
PCR confirmation of site-specific integration in the exconjugants. (**a**) Integration of pHG4 into the chromosome. (**b**) PCR (using primers TG1-attL-Am-for/rev) of the expected TG1 *attL*-containing fragment from *A. marina* DSM45569:pHG4. M: Fast DNA Ladder. Colonies 1 to 6 are independent exconjugants. (**c**) Integration of pJH1R4 into the chromosome. (**d**) PCR (using primers R4-attL-Am-for/rev) of the expected R4 *attL*-containing fragment from *A. marina* DSM45569:pJH1R4. M: Fast DNA Ladder. Colonies 1 to 6 are independent exconjugants

**Fig. 4:**
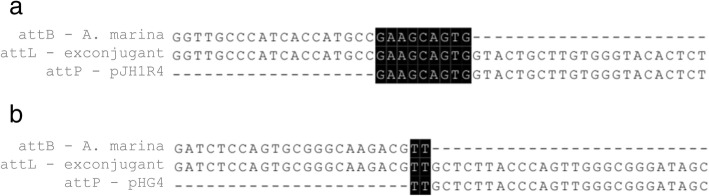
The insertion sites of R4 (**a**) and TG1 (**b**) integration plasmids in *A. marina* DSM45569. Sequencing (using primers R4-attL-Am-for or TG1-attL-Am-for) of PCR products containing *attL* from exconjugants validated the site-specific recombination of the R4 and TG1 *attB* sites in *A. marina* DSM45569 after the introduction of pHG4 or pJH1R4, respectively

*A. marina* DSM45569 is unable to grow in the presence of apramycin, so integrating plasmids pHG4 and pJH1R4, containing the apramycin resistance determinant *aac(3)IV*, were constructed. Following the standard *Streptomyces* conjugation protocol (see Methods), a frequency of approximately 160 exconjugants/10^8^ spores was obtained for the transfer of pHG4 (encoding TG1 integrase), while the conjugation efficiency of pJH1R4 (R4 integrase) was only 20 exconjugants/10^8^ spores (Table 3). For each integration, six exconjugants were picked at random and streaked on SM (soya mannitol) agar containing apramycin. Genomic DNA was then prepared and used as the template in PCR reactions, in which the primer pairs of TG1-attL-Am-for/rev and R4-attL-Am-for/rev were used to test for the occurrence of recombination at the expected TG1 and R4 *attB* sites (Fig. 3). All PCR reactions using exconjugants as templates gave the expected band sizes. Sequencing (GATC, Germany) of the PCR products with the primers TG1-attL-Am-for and R4-attL-Am-for confirmed that the plasmids had integrated into the predicted *attB* sites for TG1 or R4 integrase within *A. marina* DSM45569 (Fig. 4).

**Table 3:** Conjugation efficiency of pHG4 and pJH1R4 in different species

Exconjugants/10^8^ spores	pHG4	pJH1R4
*A. marina*	160	20
*Streptomyces coelicolor*	1.47 × 10^3^	3.28 × 10^4^
*Streptomyces . lividans*	1.56 × 10^3^	3.33 × 10^4^

## Discussion

The lack of effective genetic engineering tools is considered one of the greatest hindrances in the search for new natural products from rare Actinomycetes [33–35]. Previous studies in rare Actinomycete species have focused mainly on the use of the well-characterised ϕC31-based integration vectors, and have mostly overlooked tools based on other phage integrases [36–38]. Additionally, the easy-handling conjugation methods used widely in *Streptomyces* gene transfer have shown little success in rare Actinomycetes, including species in the genus *Amycolatopsis*, so direct transformation with plasmids [39–41], or electroporation, has been the long-preferred method of gene transfer for species in this genus [5, 42–44]. However, the growing interest in the use of serine integrases for synthetic biology applications [10] has led to further research into expanding the pool of available enzymes and their potentials as genetic tools [45–47]. Therefore, within this study, we explored whether integrating vectors based on eight serine integrases could be employed for the genetic engineering of *A. marina* DSM45569. Sequence analysis of the *A. marina* DSM45569 genome identified close matches to the *attB* sites used by TG1 and R4 integrases. Although conjugation frequencies were relatively low, integrating plasmids based on the TG1 and R4 recombination systems have been successfully integrated into the expected *attB* sites in *A. marina* DSM45569. Conservation between the *attB* sites for TG1 and R4 in a number of *Amycolatopsis* species is high, suggesting that plasmids with the integration systems from these phages should be widely useful in this genus, including the species which have garnered much interest as natural product producers, such as *Amycolatopsis balhimycina* [40], *Amycolatopsis orientalis* [20], and *A. mediterranei* [39].

As is common with serine integrase-mediated recombination, the *attB* sites in *A. marina* are located within open reading frames and potentially disrupt the gene. The TG1 *attB*^*Am*^ site is located within a gene predicted to encode a putative succinyldiaminopimelate transaminase (WP_091671332.1), and the R4 *attB*^*Am*^ site is located within a gene predicted to code for a fatty-acyl-CoA synthase (SFB62308.1). Compared to the wild-type (unintegrated) strain, the strains with integrated pHG4 or pJH1R4 did not show any difference in growth. However, further study is required to investigate the effects of TG1 or R4 plasmid recombination on both primary and secondary metabolism as, for example, the integration of ϕC31 integrase-based plasmids has been shown to have pleiotropic effects on bacterial physiology [48].

Currently, the following methods have been used to establish a gene transfer system in *Amycolatopsis* species: protoplast transformation, direct transformation of mycelia, electroporation, electroduction, and conjugation [41]. Among them, direct transformation and electroporation are most popular. While for the conjugation methods which have been widely used in *Streptomyces* species, there are few publications on conjugative transfer of vectors based on serine integrases in *Amycolatopsis*: pSET152 based on ϕC31 into *A. japonicum* MG417-CF17 (conjugation frequency = 2.4 × 10^4^ exconjugants/10^8^ spores) [19] and pDZL802 based on ϕBT1 into *A. mediterranei* U32 (4 × 10^3^ exconjugants/10^8^ spores) [21]. In this study, we successfully integrated plasmids into the *attB* sites for TG1 and R4 integrases by conjugation, which supplements the potential gene transfer methods that could be used in the genus *Amycolatopsis*, broadens the applicability of gene transfer systems except for the ones based on ϕC31 and ϕBT1 in previous publications, and will definitely facilitate the genetic manipulation of *Amycolatopsis*. Although the recombination efficiencies were lower for TG1 and R4, the conjugation conditions could be further optimised to achieve better conjugation results, or the application of integration based vectors for direct transformation of mycelia could be explored since the integrative vectors, for example, pMEA100 [39] and pMEA300 [49], used in direct transformation are based on integrase and corresponding *attP* site as well.

## Conclusions

In conclusion, we have identified highly conserved sequences of the *attB* sites for TG1 and R4 integrases within the genus *Amycolatopsis* and demonstrated their use in conjugative DNA transfer. The *A. marina* DSM45569 *attB* sites showed slightly lower recombination efficiencies in vitro than the previously identified *attB* sites from *Streptomyces* spp. However, this slight reduction is not enough by itself to explain the order of magnitude reductions in conjugation frequencies observed with *A. marina* compared to *Streptomyces* spp. (Table 3). Optimising conjugation conditions could increase the conjugation frequencies further. Alternatively, efficiently used *attB* sites for the widely used vectors, such as those based on ϕC31 *int/attP* could be incorporated into the *Amylcolatopsis* genome using TG1 or R4 integrating plasmids as described here. In short, this work shows that integrative vectors are viable and promising tools for the genetic engineering of rare Actinomycetes.

## Methods

### Bacterial strains and culture conditions

Plasmid propagation and subcloning was conducted using *E. coli* Top10 (F- *mcrA* Δ(*mrr-hsdRMS-mcrBC*) φ80*lacZ*ΔM15 *ΔlacX74 nupG recA1 araD139* Δ(*ara-leu)7697 galE15 galK16 rpsL*(Str^R^) *endA1* λ^−^). Plasmid conjugations from *E. coli* to *A. marina* DSM45569 were carried out using *E. coli* ET12567(pUZ8002) containing the plasmid to be transferred as the donor [50, 51], and conjugations from *E. coli* to *S. coelicolor* and *S. lividans* were used as control. *E. coli* strains were grown in Luria-Bertani broth (LB) or on LB agar at 37 °C.

*A. marina* DSM45569 was purchased from the German Collection of Microorganisms and Cell Cultures (DSMZ, Germany), and maintained on SM agar plates at 30 °C. Harvested spores were maintained long-term in 20% glycerol at -80 °C. Conjugations were plated on SM agar plates containing 10 mM MgCl_2,_ and ISP2 medium [52] was used for the preparation of genomic DNA [51].

### DNA manipulation

*E. coli* transformation and gel electrophoresis were carried out as described previously [53]. Genomic DNA preparation from *Streptomyces* was performed following the salting out procedure in the *Streptomyces* manual [51]. Plasmids from *E. coli* were prepared using QIAprep® Spin Miniprep Kit (Qiagen, Germany) following the manufacturer’s instructions. Polymerase Chain Reaction (PCR) was carried out using Phusion® High-Fidelity DNA Polymerase (NEB, USA) according to the manufacturer’s instructions. The primers used in this study are listed in Table 1. DNA samples were purified by the QIAquick Gel Extraction Kit (Qiagen, Germany).

### Plasmid construction

**Fig. 5:**
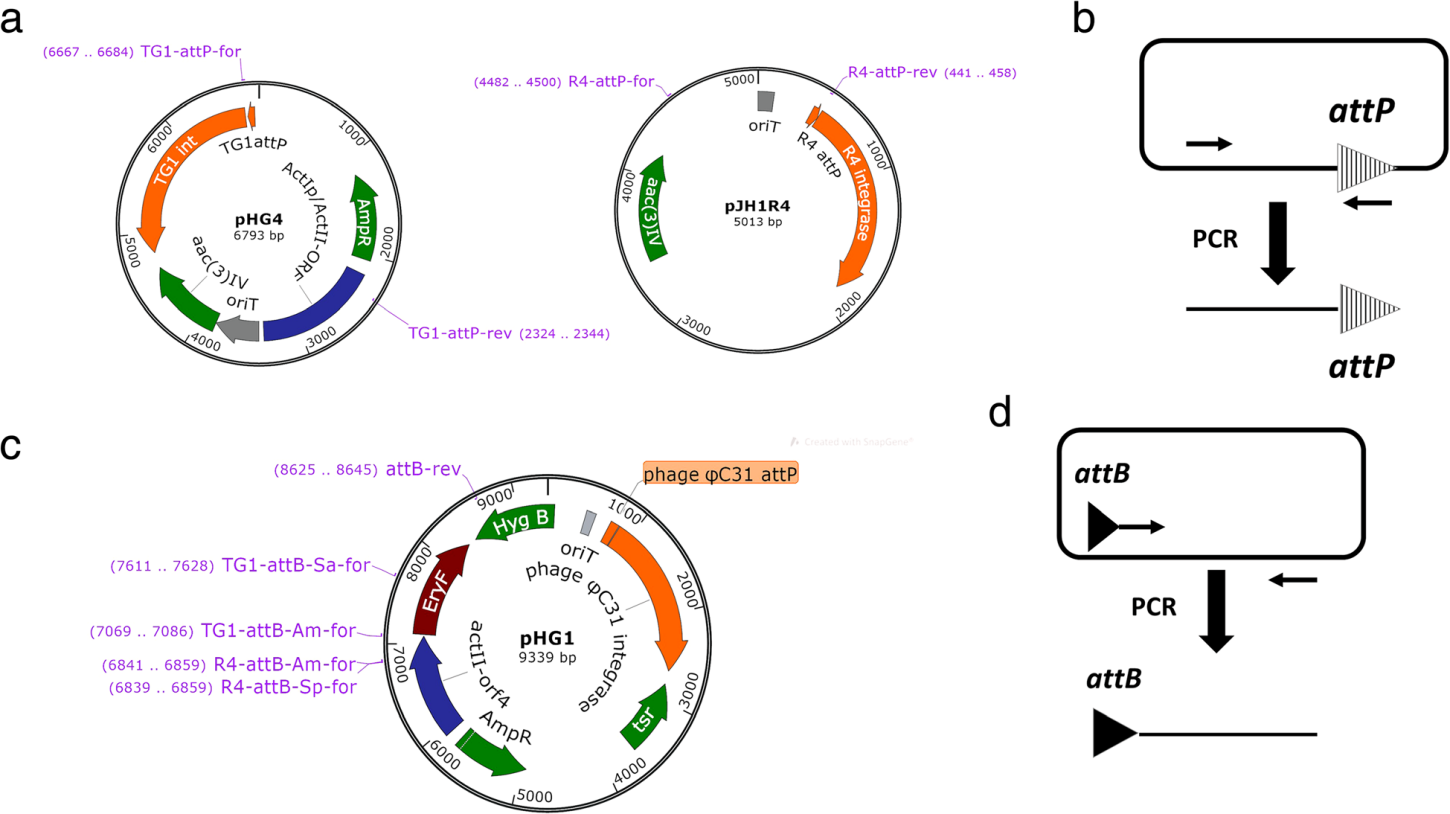
Plasmids used in this study. The primer binding sites are indicated. (**a**) Integrating plasmids pHG4 and pJH1R4; (**b**) DNA substrate *attP*s amplified from pHG4 and pJH1R4; (**c**) PCR template plasmid pHG1; (**d**) DNA substrate *attB*s amplified from pHG1

The integrating plasmid pHG4 contains the TG1 *int*/*attP* locus and the apramycin-resistance gene (*aac(3)IV*) for selection (Fig. 5a). The fragment containing *oriT*, *aac(3)IV* and TG1 *int*/*attP* was amplified from plasmid pBF20 [54] using the primer pair pHG4-for/pHG4-rev. The fragment was joined via In-Fusion cloning to the 3344 bp HindIII-PacI fragment from pBF22 [54] (containing the *E. coli* plasmid replication origin, the *bla* gene encoding resistance to ampicillin and the *actII-orf4/act1p* expression cassette) to form the plasmid pHG4.

To construct the integrating plasmid pJH1R4 (Fig. 5a), pSET152 [55] was cut with AatII and PvuI to remove the ϕC31 *attP* site and integrase gene. R4 phage lysate was used as the template in a PCR with the primers pJH1R4-for and pJH1R4-rev to amplify the R4 *attP* site and integrase coding region. The PCR product was joined to the AatII-PvuI fragment from pSET152 via In-Fusion cloning.

The plasmid pHG1 (Fig. 5c) was used as the template in PCR to amplify *attB*-containing sequences (Fig. 5d) for in vitro recombination assays. This plasmid was initially constructed for the expression of *EryF*. The *eryF* gene was amplified from *Saccharopolyspora erythraea* BIOT-0666 genomic DNA using the primer pair pHG1A-for/pHG1A-rev, and inserted by In-Fusion cloning into pBF20 [54] cut with NheI and PacI to form the plasmid pHG1A. The 3785 bp fragment containing the ϕC31 *int*/*attP* and hygromycin resistance gene was amplified from plasmid pBF27C [54], using the primer pair pHG1-for and pHG1-rev. Plasmid pHG1A was digested with XbaI and NheI, and the 5668 bp fragment was ligated with the 3785 bp PCR fragment from pBF27C by In-Fusion cloning to give the plasmid pHG1.

### In vitro recombination assays

In vitro recombination assays were performed using PCR-amplified DNA fragments containing the *attB* and *attP* attachment sites located at the ends. Recombination between the *attP* and *attB* sites joined the two fragments to give a product whose length was almost the sum of the substrates (Fig. 2a). To generate the *attB*-containing substrates, the forward primer, TG1-attB-Am-for, contained the closest match in the *A. marina* genome to the characterised TG1 *attB* site from *S. avermitilis,* TG1 *attB*^*Sa*^ [30] (Fig. 1). TG1-attB-Am-for also had a sequence identical to the 3′ end of the act1p element from plasmid pHG1, which was used as a template for PCR (Fig. 5c). Similarly, the forward primer R4-attB-Am-for contained the closest match in the *A. marina* genome to the characterised R4 *attB* site from *S. parvulus,* R4 *attB*^*Sp*^ [32] (Fig. 1). R4-attB-Am-for also had a sequence identical to the 3′ end of ActII-orf4 element from the template plasmid pHG1 (Fig. 5d). Forward primers TG1-attB-Sa-for and R4-attB-Sp-for were used to create positive control recombination substrates containing the TG1 and R4 *attB* sites originally found in *S. avermitilis* [30] and *S. parvulus* [32] respectively. The reverse primer used to generate all the *attB*-containing substrates (attB-rev) was located within the *hyg* gene of pHG1; the amplified products were 1627 bp (TG1 *attB*^Am^), 1035 bp (TG1 *attB*^Sa^), 1854 bp (R4 *attB*^Am^) and 1855 bp (R4 *attB*^Sp^). The DNA fragments containing the *attP* sites were prepared as follows; the TG1-*attP* fragment (2471 bp) was amplified using the primer pair TG1-attP-for/TG1-attP-rev with pHG4 as the template, and the R4-*attP* fragment (990 bp) was amplified using the primer pair R4-attP-for/R4-attP-rev with pJH1R4 as the template (Fig. 5b). Note that other than the *attB* and *attP* sites, none of the substrates contained any DNA that should interact specifically with the integrases. Moreover, each fragment was designed to be easily identifiable by molecular weight.

The integrases were purified as described previously [27, 56]. All recombination reactions were in 20 μl final volume. Recombination reactions of TG1 substrates were carried out in TG1 RxE buffer (20 mM Tris [pH 7.5], 25 mM NaCl, 1 mM dithiothreitol [DTT], 10 mM spermidine, 10 mM EDTA, 0.1 mg/ml bovine serum albumin [BSA]) [57], and recombination reactions of R4 substrates were carried out in buffer containing 20 mM Tris-HCl (pH 7.5), 50 mM NaCl, 10 mM spermidine, 5 mM CaCl_2_ and 50 mM DTT [27]. Integrase was added at the concentrations indicated. Recombination substrates were used at 50 ng each per reaction. Reactions were incubated at 30 °C overnight and then heated (10 min, 75 °C) to denature integrase. The reaction mixtures were loaded on a 0.8% agarose gel in Tris/Borate/EDTA (TBE) buffer (90 mM Tris base, 90 mM boric acid and 2 mM EDTA) containing ethidium bromide for electrophoretic separation.

## Abbreviations

BSA: Bovine serum albumin
PCR: Polymerase chain reaction
RDF: Recombination directionality factor
SM: Soya Mannitol
TBE: Tris/Borate/EDTA
